# Frequency of IDH1 mutation in adult-type diffuse astrocytic gliomas in a tertiary hospital in Kenya

**DOI:** 10.3389/fmed.2024.1305714

**Published:** 2024-02-19

**Authors:** Samuel Gakinya, Anderson Mutuiri, Timothy Onyuma, Beverly Cheserem, Edwin Mogere

**Affiliations:** ^1^Department of Pathology, Aga Khan University, Nairobi, Kenya; ^2^Kenyatta National Hospital, Nairobi, Kenya

**Keywords:** glioma, IDH1, astrocytoma, Kenya, frequency, Africa

## Abstract

The 2021 WHO classification of gliomas has separated gliomas based on their IDH mutation status, reflecting differences in their pathogenesis and clinical characteristics. There is a paucity of data on the prevalence of IDH mutations in gliomas in this region. This study aimed to determine the frequency of the IDH1 mutation in adult-type diffuse astrocytic gliomas in a tertiary hospital in Kenya. Approximately half of the gliomas were positive for the IDH1 mutation, with a slight male predominance. Our study provides crucial insights into the frequency of IDH1 mutations in gliomas in Kenya.

## Introduction

Gliomas are the most common primary intracranial brain tumors in adults (1). Over the years, there have been changes in the classification of gliomas, driven by an increasing body of knowledge on their pathogenesis. The 2021 WHO classification of central nervous system tumours 5th edition classifies diffuse adult type gliomas into three categories: astrocytoma IDH-mutant (grade 2–4), oligodendroglioma IDH-mutant and 1p/19q-codeleted (grade 2–3) and glioblastoma IDH-wildtype (grade 4) (2). This classification and grading are based on morphological and molecular characteristics, including IDH mutation status. This classification, unlike the previous, has separated IDH-mutant astrocytic gliomas from IDH wild-type gliomas, reflecting their different pathogenesis, clinical–pathological characteristics, and prognosis.

Isocitrate dehydrogenase (IDH) is a Krebs cycle enzyme that catalyzes the oxidative decarboxylation of isocitrate to alpha-ketoglutarate (α-KG). This reaction generates NADPH and NADH, which play an important role in cellular protection against oxidative stress (3). The IDH mutation results in the gain of a new enzymatic activity in which alpha-ketoglutarate is reduced to D-2-hydroxyglutarate (D-2HG), an oncometabolite that causes hypermethylation of the CpG islands in the genome. This silences the expression of genes important in cellular differentiation, which results in oncogenic transformation (3). The most frequent IDH mutation in gliomas is *IDH1*:c.395G > A p.R132H, accounting for 83–91% of the mutations (4–6).

In addition to its role in the pathogenesis of IDH-mutant gliomas, IDH mutation status is a biomarker of predictive and prognostic importance (7). IDH-mutant patients have better overall survival and progression-free survival, irrespective of grade (8). IDH mutation is also predictive of response to vorasidenib in low-grade gliomas (9).

While there is a lot of literature on IDH mutations and gliomas from developed countries, there is a paucity of data on the same from Africa. This is partly due to the lack of resources for the molecular tests required to detect mutations. IDH mutation status can be determined using different methods, the most common being sequencing and immunohistochemistry (10, 11). Sequencing is a more robust method, as it can detect all the mutations in both IDH1 and IDH2. It is, however, expensive and not readily available in developing countries. Immunohistochemistry, on the contrary, is affordable and more accessible in developing countries but is limited in the number of mutations that it can detect. Currently, the only mutation that can be determined using immunohistochemistry is the *IDH1*:c.395G > A p.R132H mutation, which is the most common.

The objective of this study was to identify the frequency of IDH R132H in adult-type diffuse astrocytic gliomas using immunohistochemistry as well as describe their clinical and pathological characteristics, including age, gender, location, and grade.

## Methodology

This was a retrospective study conducted at the Aga Khan University Hospital Nairobi laboratory, a College of American Pathologists (CAP)-accredited laboratory. All cases diagnosed as diffuse astrocytoma grade II (IDH1 mutant and NOS), anaplastic astrocytoma grade III (IDH1 mutant and NOS), glioblastoma (IDH1 mutant and NOS), and astrocytoma IDH1-mutant grades 2–4 from January 2019 to March 2023 were identified from the laboratory information system. For each case, the age, gender, tumor type and grade, location, and IDH1 status were recorded.

**Figure 1:**
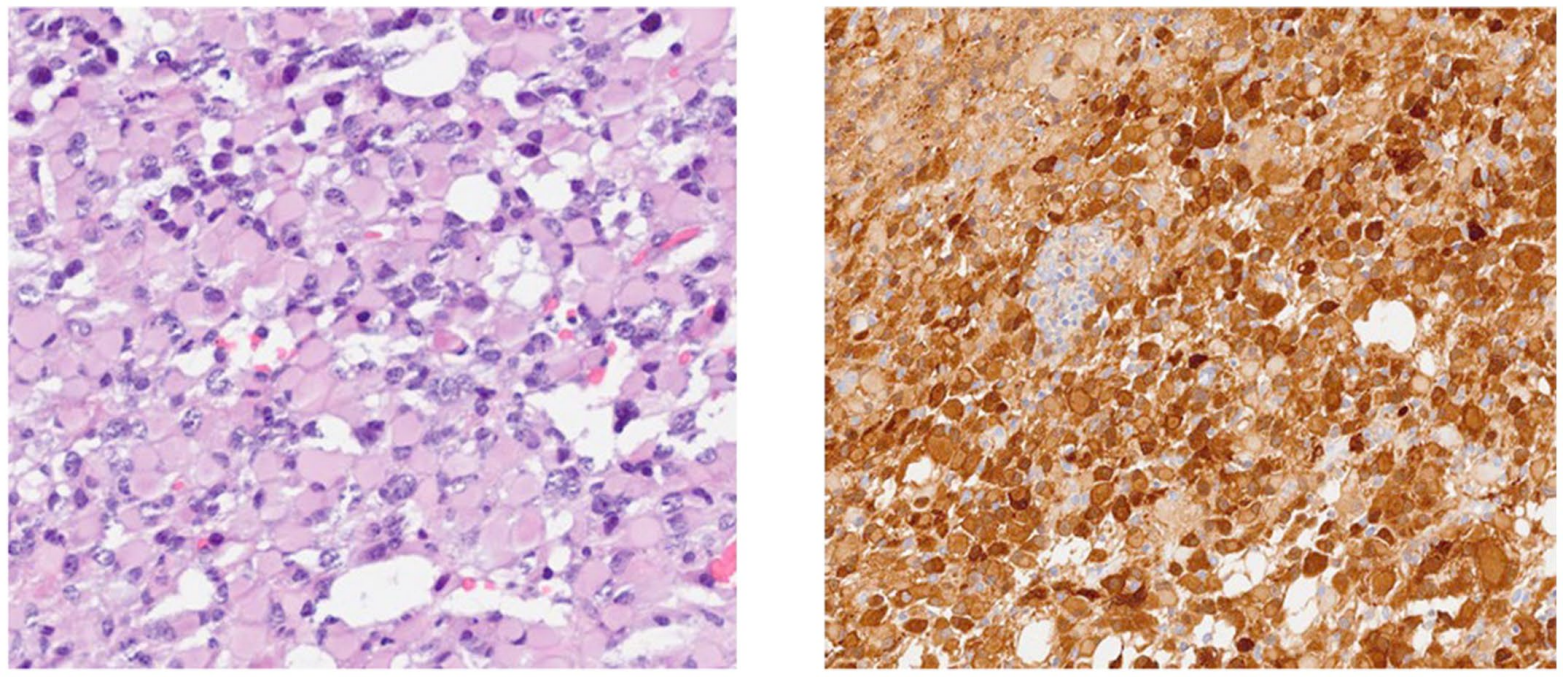
IDH1-positive glioma.

**Figure 2:**
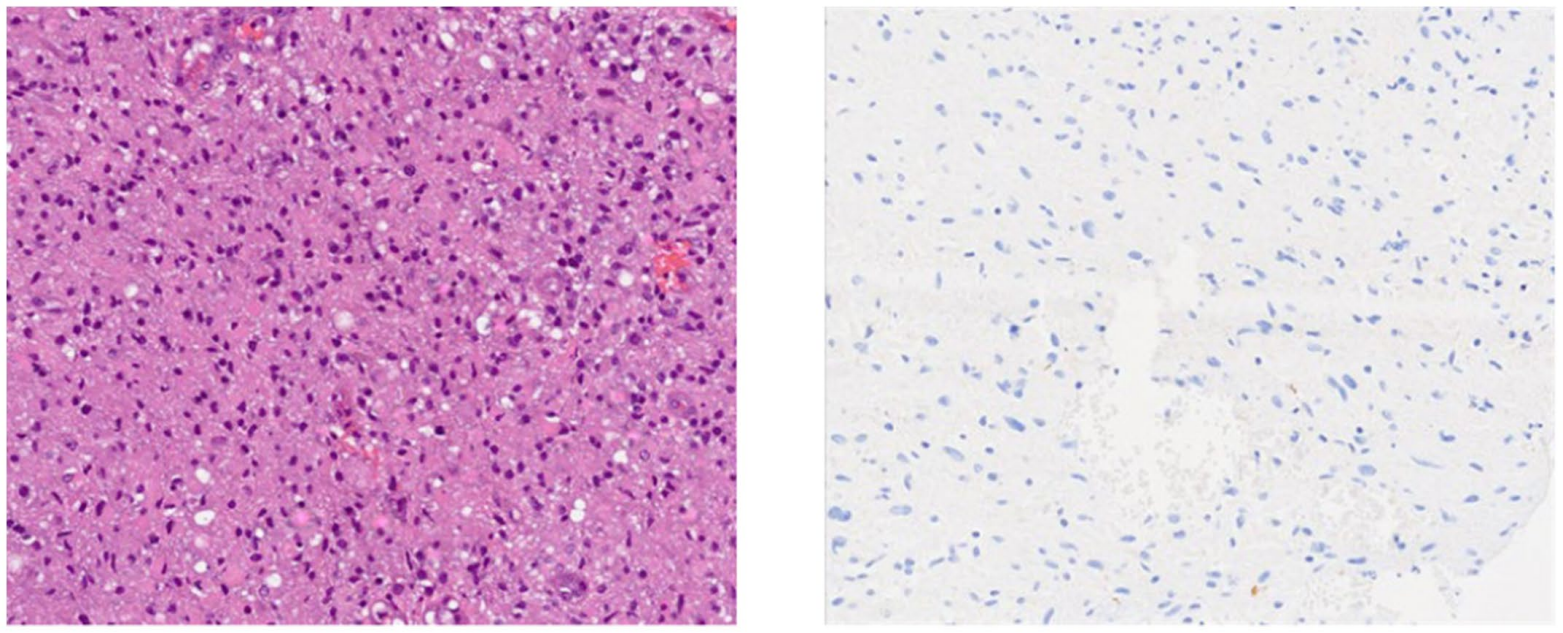
IDH1-negative glioma.

The standard operating procedure for handling diffuse gliomas in the laboratory includes an initial examination of the hematoxylin- and eosin-stained slides. For tumors whose morphology is suggestive of an astrocytic or oligodendroglial neoplasm, immunohistochemistry for GFAP, OLIG2, IDH1, and ATRX is performed. Tumors are categorized as astrocytic based on characteristic morphology and positivity for GFAP and/or OLIG2, with or without ATRX loss and IDH1 status. IDH1 status is reported as mutant (positive) or negative, depending on the staining (Figures 1, 2).

The grading is based on the morphologic characteristics, including anaplasia, mitotic activity, endothelial proliferation, and necrosis. Oligodendrogliomas are differentiated from astrocytomas based on their characteristic morphology, absence of GFAP staining, except in minigemistocytes and gliofibrillary oligodendrocytes (12, 13), and retained ATRX, IDH1, and OLIG2 positivity. Other molecular investigations are not available.

Ethical approval was granted by the Aga Khan University research ethics committee.

## Results

### Gender and age distribution of gliomas

A total of 62 glioma cases were identified, of which 54.8% were men and 45.2% were women. The median age was 46 years for both genders, while the range was 18–90 years for men and 18–80 years for women (Table 1).

**Table 1:** Gender and age distribution of gliomas.

Gender		*N*	Percent	Median age (Years)
	Male	34	54.8	46
	Female	28	45.2	46
	Total	62	100.0	

### Gliomas and IDH

A total of 51.6% of the gliomas were positive for IDH1, while 48.4% were negative (Table 2).

**Table 2:** Frequency of IDH1-positive gliomas.

IDH1 Status		*N*	Percent	Median age (Years)
	Positive	32	51.6	42
	Negative	30	48.4	49
	Total	62	100.0	

**Figure 3:**
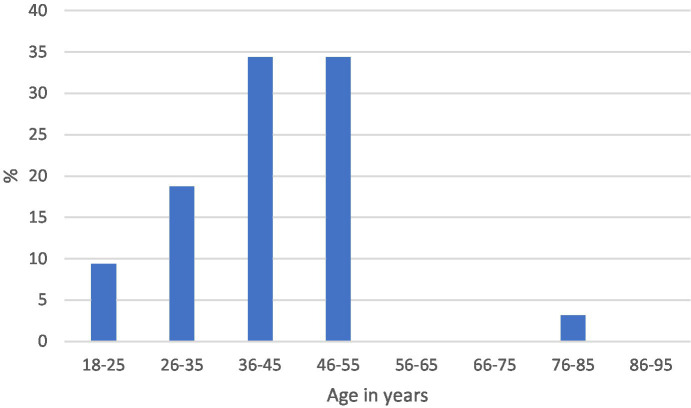
Age distribution of IDH1-positive gliomas.

**Figure 4:**
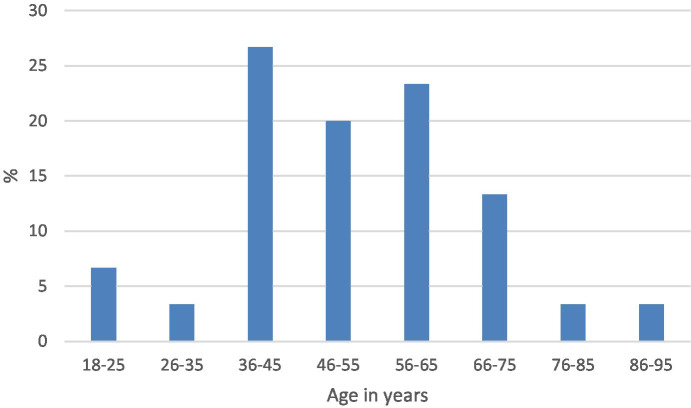
Age distribution of IDH1-negative gliomas.

The median age and range were 42 years (18–82) and 49 years (18–90) in the positive and negative categories, respectively. The distribution by age in each of the categories is shown in Figures 3, 4.

### Astrocytoma IDH1 mutant

Based on their histological characteristics, the IDH1-positive tumors were categorized according to the 2021 WHO classification into astrocytoma IDH1-mutant grades 2–4. The most frequent was astrocytoma IDH1-mutant grade 4. Astrocytoma IDH1-mutant grade 4 had a higher median age of 51 years, while grades 2 and 3 were comparable with a median age of 39 and 38 years, respectively (Table 3).

**Table 3:** Grade and age distribution of astrocytoma IDH1-mutant gliomas.

Tumor grade	*N*	Percent	Median age (years)
Astrocytoma IDH1-mutant grade 2	11	34.4	39
Astrocytoma IDH1-mutant grade 3	7	21.8	38
Astrocytoma IDH1-mutant grade 4	14	43.8	51
	32	100.0	

### IDH1-negative gliomas

The IDH1-negative tumors were assigned a category and graded based on their histological features, and a prefix NOS (not otherwise specified) was included as molecular diagnosis was not performed to rule out the less common IDH mutations. The categories were glioblastoma grade IV NOS, anaplastic astrocytoma grade III NOS, and diffuse astrocytoma grade II NOS. The most frequent type was glioblastoma NOS, with a median age of 56 years (Table 4).

**Table 4:** Grade and age distribution of IDH1-negative gliomas.

Tumor grade	*N*	Percent	Median age (years)
Diffuse astrocytoma grade II NOS	2	6.7	42
Anaplastic astrocytoma grade III NOS	2	6.7	23
Glioblastoma grade IV	26	86.6	56
	30	100.0	

### Gliomas and location

The data on the tumor location were not available for the majority (51.6%) of the tumors. Of the ones with available data, supratentorial was the most common location (left hemisphere 25.8% and right hemisphere 21.0%). Only 1.6% of the tumors were infratentorial.

### ATRX and IDH1

Of the 62 cases, only 28 had ATRX staining carried out. Of these, 10 (35.7%) had lost ATRX expression, while 18 (64.3%) had retained expression. Of the cases with lost expression, all (100%) were IDH1 positive. The median age (range) of the ATRX-mutated/IDH-mutated patients was 34 years (18–48).

## Discussion

The 2021 WHO classification of central nervous system tumors fifth edition has classified gliomas based on their morphology and molecular characteristics (2). Of the molecular characteristics, the IDH mutation is a defining characteristic of astrocytomas. IDH-mutant astrocytic tumors are now recognized as a different entity from the IDH wild type and have been categorized as astrocytoma IDH-mutant grades 2–4. This distinction not only reflects the different biological characteristics of these tumors but also their prognosis and response to therapy. This classification, in addition, recognizes pediatric gliomas as separate from adult gliomas, acknowledging their different tumor biology. This study therefore included only adult patients to give an accurate frequency of IDH-mutant diffuse astrocytic neoplasms.

In our study, the gender distributions of diffuse astrocytic tumors showed a slight male predominance; similar to what is in the literature (14). There was an almost equal distribution in the frequency of IDH1-positive and IDH1-negative astrocytomas. This frequency of IDH mutation is lower than that reported in studies from Europe (4, 15) while higher than that reported in studies from Asia (16–18). These differences might, however, be attributed to different methodologies of IDH testing as well as changes in the nomenclature of gliomas over time.

The majority of the IDH1 positive tumors were grade 4. Grade 4 tumors were common in patients in their fifth decade, while grades 2 and 3 were common in patients in their third decade. These results are similar to other studies that have shown most IDH-mutant astrocytoma is grade 4 and occurs in older patients, while grade 2 and 3 tumors have a similar age distribution in younger patients (4, 5, 15, 19). In the current WHO classification, the criteria for grade 4 adult diffuse astrocytoma not only include the presence of microvascular proliferation and/or necrosis but also homozygous deletion of CDKN2A and/or CDKN2B. Some grade 4 tumors may therefore have been graded as grade 2 or 3 in this study, as this was not performed.

For the IDH1 negative tumors, the majority of the tumors were grade 4 and occurred in patients in their fifth decade. This is similar to what has been described in other studies (15, 20).

The supratentorial region was the most common tumor location. Most of the cases, however, did not have this information.

Similar to other studies (21), ARTX loss was associated with the IDH1 mutation. In this study, all the ATRX-negative cases were IDH1 mutants. Our data on ATRX are, however, small, and data on location are not available for the majority of the cases; hence, we need to carry out future studies on the same.

In conclusion, the frequency of the IDH1R132H mutation in adult-type diffuse gliomas is 51% in our institution, with a slight male predominance. Among both the IDH1 R132H-mutant astrocytomas and the IDH1R132H-negative diffuse astrocytomas, the distribution by grade and age is as described in other studies. While not carrying out all the molecular diagnostic tests as described in the current classification is a limitation, the study provides important baseline information on the frequency of IDH mutation in adult diffuse astrocytoma in the country, as no studies have previously been carried out in the region.

## Data availability statement

The raw data supporting the conclusions of this article will be made available by the authors, without undue reservation.

## Ethics statement

The studies involving humans were approved by the Aga Khan University, Nairobi Institutional Scientific and Ethics Review Committee (ISERC). The studies were conducted in accordance with the local legislation and institutional requirements. The ethics committee/institutional review board waived the requirement of written informed consent for participation from the participants or the participants’ legal guardians/next of kin because the study is an audit which only looked at the histology results of the glioma samples and extracted the demographic data, histologic diagnosis, and IDH1 status. No intervention was performed. The patient identity was anonymized.

## Author contributions

SG: Writing – original draft, Methodology. AM: Writing – review & editing, Methodology. TO: Formal analysis, Writing – review & editing. BC: Conceptualization, Writing – review & editing. EM: Conceptualization, Writing – review & editing.

## References

[1] Ostrom QT Bauchet L Davis FG Deltour I Fisher JL Langer CE. (2023). The epidemiology of glioma in adults: a “state of the science” review. Available at: https://academic.oup.com/neuro-oncology/article/16/7/896/1927249 10.1093/neuonc/nou087 PMC4057143 24842956

[2] Louis DN Perry A Wesseling P Brat DJ Cree IA Figarella-Branger D. The 2021 WHO classification of tumors of the central nervous system: a summary. Neuro-Oncology. (2021) 23:1231–51. doi: 10.1093/neuonc/noab106, PMID: 34185076 PMC8328013

[3] Waitkus MS Diplas BH Yan H. Isocitrate dehydrogenase mutations in gliomas. Neuro-Oncology. (2016) 18:16–26. doi: 10.1093/neuonc/nov136, PMID: 26188014 PMC4677412

[4] Balss J Meyer J Mueller W Korshunov A Hartmann C von Deimling A. Analysis of the IDH1 codon 132 mutation in brain tumors. Acta Neuropathol. (2008) 116:597–602. doi: 10.1007/s00401-008-0455-2 18985363

[5] Bleeker FE Lamba S Leenstra S Troost D Hulsebos T Vandertop WP. IDH1 mutations at residue p.R132 (IDH1R132) occur frequently in high-grade gliomas but not in other solid tumors. Hum Mutat. (2009) 30:7–11. doi: 10.1002/humu.20937, PMID: 19117336

[6] Watanabe T Nobusawa S Kleihues P Ohgaki H. IDH1 mutations are early events in the development of Astrocytomas and Oligodendrogliomas. Am J Pathol. (2009) 174:1149–53. doi: 10.2353/ajpath.2009.080958, PMID: 19246647 PMC2671348

[7] Śledzińska P Bebyn MG Furtak J Kowalewski J Lewandowska MA. Prognostic and predictive biomarkers in gliomas. Int J Mol Sci. (2021) 22:10373. doi: 10.3390/ijms221910373, PMID: 34638714 PMC8508830

[8] Zou P Xu H Chen P Yan Q Zhao L Zhao P. IDH1/IDH2 mutations define the prognosis and molecular profiles of patients with gliomas: a meta-analysis. PLoS One. (2013) 8:e68782. doi: 10.1371/journal.pone.0068782, PMID: 23894344 PMC3718803

[9] Mellinghoff IK van den Bent MJ Blumenthal DT Touat M Peters KB Clarke J. Vorasidenib in IDH1-or IDH2-mutant low-grade glioma. N Engl J Med. (2023) 389:589–601. doi: 10.1056/NEJMoa2304194, PMID: 37272516 PMC11445763

[10] Brat DJ Aldape K Bridge JA Canoll P Colman H Hameed MR. Molecular biomarker testing for the diagnosis of diffuse gliomas: guideline from the College of American Pathologists in collaboration with the American Association of Neuropathologists, Association for Molecular Pathology, and Society for Neuro-Oncology. Arch Pathol Lab Med. (2022) 146:547–74. doi: 10.5858/arpa.2021-0295-CP 35175291 PMC9311267

[11] Śledzińska P Bebyn M Szczerba E Furtak J Harat M Olszewska N. Glioma 2021 WHO classification: the superiority of NGS over IHC in routine diagnostics. Mol Diagn Ther. (2022) 26:699:–713. doi: 10.1007/s40291-022-00612-3, PMID: 36053463 PMC9626418

[12] Herpers MJHM Budka H. Glial fibrillary acidic protein (GFAP) in oligodendroglial tumors: gliofibrillary oligodendroglioma and transitional oligoastrocytoma as subtypes of oligodendroglioma. Acta Neuropathol. (1984) 64:265–72. doi: 10.1007/BF00690392, PMID: 6391068

[13] Reifenberger G Szymàs J Wechsler W. Differential expression of glial-and neuronal-associated antigens in human tumors of the central and peripheral nervous system. Acta Neuropathol. (1987) 74:105–23. doi: 10.1007/BF00692841, PMID: 3314309

[14] WHO (2023). BlueBooksOnline. Available at: https://tumourclassification.iarc.who.int/chapters/45/3 (Accessed August 24, 2023).

[15] Hartmann C Meyer J Balss J Capper D Mueller W Christians A. Type and frequency of IDH1 and IDH2 mutations are related to astrocytic and oligodendroglial differentiation and age: a study of 1,010 diffuse gliomas. Acta Neuropathol. (2009) 118:469–74. doi: 10.1007/s00401-009-0561-9, PMID: 19554337

[16] Mukasa A Takayanagi S Saito K Shibahara J Tabei Y Furuya K. Significance of IDH mutations varies with tumor histology, grade, and genetics in Japanese glioma patients. Cancer Sci. (2012) 103:587:–592. doi: 10.1111/j.1349-7006.2011.02175.x, PMID: 22136423 PMC7713609

[17] Malueka RG Dwianingsih EK Bayuangga HF Panggabean AS Argo IW Donurizki AD. Clinicopathological features and prognosis of Indonesian patients with gliomas with IDH mutation: insights into its significance in a southeast Asian population. Asian Pac J Cancer Prev. (2020) 21:2287:–2295. doi: 10.31557/APJCP.2020.21.8.2287, PMID: 32856857 PMC7771930

[18] Cho U Yang SH Yoo C. Estimation of the occurrence rates of IDH1 and IDH2 mutations in gliomas and the reconsideration of IDH-wildtype anaplastic astrocytomas: an institutional experience. J Int Med Res. (2021) 49:030006052110192. doi: 10.1177/03000605211019258 PMC8236789 34162262

[19] Reuss DE Mamatjan Y Schrimpf D Capper D Hovestadt V Kratz A. IDH mutant diffuse and anaplastic astrocytomas have similar age at presentation and little difference in survival: a grading problem for WHO. Acta Neuropathol. (2015) 129:867–73. doi: 10.1007/s00401-015-1438-8, PMID: 25962792 PMC4500039

[20] Robinson C Kleinschmidt-Demasters BK (2023). IDH1-mutation in diffuse gliomas in persons age 55 years and over. Available at: https://academic.oup.com/jnen/article/76/2/151/2936785 (Accessed July 25, 2023). 10.1093/jnen/nlw112 28110298

[21] Ikemura M Shibahara J Mukasa A Takayanagi S Aihara K Saito N. Utility of ATRX immunohistochemistry in diagnosis of adult diffuse gliomas. Histopathology. (2016) 69:260–7. doi: 10.1111/his.12927 26741321

